# Associated factors of first-birth interval among women in reproductive age, addressing maternal and child health

**DOI:** 10.1186/s12978-022-01346-5

**Published:** 2022-01-29

**Authors:** Tania Dehesh, Neda Malekmohammadi, Paria Dehesh

**Affiliations:** 1 Department of Biostatistics and Epidemiology, School of Public Health, Kerman University of Medical Sciences, Kerman, Iran; 2 Modeling in Health Research Center, Institute for Futures Study in Health, Kerman University of Medical Sciences, Kerman, Iran; 3 HIV/STI Surveillance Research Center, and WHO Collaborating Center for HIV Surveillance, Institute for Futures Studies in Health, Kerman University of Medical Sciences, Kerman, Iran; 4 Department of Epidemiology, School of Public Health, Iran University of Medical Sciences, Tehran, Iran

**Keywords:** First-birth interval, Behavioral, Cox regression model

## Abstract

**Background:**

The first-birth interval directly influences family size and maternal and child mortality. The objective of this study is to investigate factors associated with the time of the first-birth after the first marriage among women in Iran.

**Methods:**

In this cross-sectional study, the first birth history of 1350 women aged 15–49 years was collected in Kerman (southern Iran) in 2018. To assess the predictor variables of the first-birth interval and calculate the adjusted hazard ratios, multivariate Cox regression was used. The P-value < 0.05 was considered as significant. The statistical analysis of data was performed using SPSS version 22.

**Results:**

The average of the first-birth interval was 2.5 ∓ 0.8 years. Woman’s age at marriage (HR 1.48, 95% CI 1.32–2.48), husband’s age at marriage (HR 1.88, 95% CI 1.62–2.03), age at the first menstruation (HR 1.53, 95% CI 1.24–2.53), being rural residents (HR 2.041, 95% CI 1.26–2.95), and having engagement period (HR 1.85, 95% CI 1.52–3.05) were associated with short first-birth interval, and woman’s BMI (HR 1.72, 95% CI 1.54–2.77), woman’s university educational level (HR 1.47, 95% CI 1.35–2.57), husband’ s university educational level (HR 1.39, 95% CI 1.32–2.51), contraception use (HR 2.28, 95% CI 1.12–2.86) and income sufficiency (HR 2.25, 95% CI 1.12–2.96) were associated with long first-birth interval.

**Conclusions:**

Being a rural resident is the most influential predictor of short first-birth interval and income sufficiency is the most influential predictor of long first-birth interval.

## Plain English summary

Total Fertility Rate, which is the average number of live births that would be born to a woman over her reproductive life if she experiences the current age-specific fertility rates throughout her lifetime. The fertility pattern can be measured by several indicators such as the first-birth interval (FBI) after marriage. FBI is defined as the duration of time spent by married couples to have their first child since the first day of marriage. Some studies have demonstrated that the length of FBI subsequently influences spacing and childbearing pattern of a family. The aim of this study is first to determine the mean of FBI and second to explore the associated factors of FBI in Kerman, southern Iran. We analyzed data for 1350 women 15–49 years old. The outcome variable was the first-birth interval (FBI). Results showed that the average of FBI was 2.5 years. There is a positive association between FBI and age of wives at marriage, first menace age, wives’ BMI, wives and husbands’ educational level, contraception use, age of husbands at marriage, residence place, income sufficiency, and having engagement period. The women and men who got married at older ages were more likely to report short FBI. It was indicated that Educated women and men and women who experience menstruation at a younger age are more likely to report longer FBI. The knowledge about importance of FBI should be more announced for couples by health services in order to help them manage their child-spacing and fertility. The government also should pay attention on FBI as an important index in determining the average age of country population.

## Introduction

Population studies include reviewing the dynamics and spacing of births [1]. Total fertility rate, which is the average number of live births that would be born to a woman over her reproductive life if she experiences the current age-specific fertility rates throughout her lifetime [2]. The fertility pattern can be measured by several indicators such as the first-birth interval (FBI) after marriage [3]. FBI is defined as the duration of time spent by married couples to have their first child since the first day of marriage [4].

Data on FBI is one of the most important fertility data due to certain reasons: first, FBI is considered an important event for most couples and so is unaffected by recall lapse; second, it is not affected by the erratic fluctuations of postpartum period [5].

The birth of the first child is recognized as the most important determinant of the fertility rates and population size, in population studies [6]. Some studies have demonstrated that the length of FBI subsequently influences spacing and childbearing pattern of a family [7–9]. The average time interval between marriage and the first birth is 2.7 years in Iran [10] 2 years in china, and 14.5 months in Tanzania [11, 12]. While delay in the first birth could have some worry for couples; early first birth, especially in unexpected and unwanted pregnancies, could lead to the same. [13].

Social factors related to the FBI include the woman’s age at marriage [14, 15], wife and husband’s education [16, 17], the continuation of woman studies, woman’s employment after marriage [18], access to contraception devices [11], wealth index [4]. There is also evidence that menstrual status can affect the FBI [19]. Menarche, or the first menstrual bleeding, is a significant event in the reproductive life of a woman. Early menarche (at the age of 11 years or below) increases the vulnerability of adolescents to negative sexual and reproductive health outcomes including early sexual initiation, early pregnancy and delivery, and sexual violence [20].

Many biological, economic, social, and cultural factors influence FBI. In developing countries such as Iran, social–behavioral factors are changing faster than other countries. These fast changes confirm the importance of investigating FBI predictors frequently. Most of Iranian studies focused on birth spacing predictors rather than FBI [21–24]. In addition, very few of them performed suitable statistical analyses for time (survival analysis). The aim of this study is first to determine the mean of FBI and second to explore the associated factors of FBI in Kerman, southern Iran with Cox regression analysis.

## Methods

This cross-sectional study was conducted with 1350 women referred to health centers of Kerman, southeast of Iran from August to December 2018. Inclusion criteria were age 15–49 years; being pregnant for the first time after marriage (first marriage) at the time of interview; being resident of Kerman since marriage, and having Iranian nationality. Exclusion criteria were having history of infertility and history of genital diseases (genital disease that can affect pregnancy, such as genital warts, gonorrhea and syphilis, etc.). The women were selected by multistage random sampling method from a list of family planning clinics. In the first stage, 10 main health centers were selected, then, in the second stage, 1350 samples were allocated based on the mean referral of pregnant women to each health center in a month. The health centers that served more pregnant women were assigned more samples. After dividing total samples between 10 main health centers, the women were randomly selected based on file numbers in each health center.

After explaining the aims of the study, women filled in the checklist about their first birth history. Illiterate women were asked the survey questions orally in the presence of a witness.

Each interview lasted for an average period of 20 min (range 15 To 25 min).

The checklist included demographic characteristics and FBI history including the couple’s age at marriage, their educational status, the women underlying diseases (blood pressure [BP], Diabetes, and kidney diseases), couple’s job status (nightly\daily), contraception use, residence place (rural\town), income sufficiency, having engagement period (means having premarital dating), and BMI.

### Predictors

The researchers of the present study explored predictors associated with the study outcome: the wife and husband age at marriage which means the number of years from birth until marriage (years old),education status of the wife and husband (Illiterate, High school and Diploma, or university education), drug use of the wife and husband which means being addicted to any types of narcotics such as: opium or heroin (yes/no), income status which means status of monthly family income based on personal perspective, the family income being completely sufficient for family needs according to family member’s expectations (Sufficient/insufficient), the women underlying diseases which means existence of any chronic disease such as blood pressure, kidney disease, or diabetes (yes/no), contraception use before the first pregnancy (yes/no), night job of the wife and husband which means having job that must be out of home at work during night (yes/no), residence place (rural/town), women's BMI (BMI is a measurement of a person’s weight with respect to his or her height), marriage type (family/not family), age at the first menstruation which means the number of years from birth to the first menstruation (years old), and having engagement period which means having premarital dating (yes/no).

### Statistical analysis

The authors of the present study used descriptive statistics (mean and standard deviation [SD]) to describe the quantitative data, and percentage and frequency for categorical data. Survival analysis is used when the outcome is a time until an event. In the current study, the event is the first birth, and the outcome is the time from marriage (first marriage) until the first child birth. Cox regression was used as the most appropriate survival model for investigating the effect of predictors on time [24]. In Cox regression, the effect of predictors on time could be determined with hazard ratio [8, 22]. The predictor with hazard ratio greater than one increases FBI. The predictor with hazard ratio less than one decreases FBI. For model building, first we fitted bivariable Cox regression between each predictor and outcome, and then the predictors with P-value less than 0.15 were chosen to be further adjusted in assessing the effect of other covariates. The researchers then created multivariable Cox regression model including all the predictors with P-value < 0.15 in bivariable models. The adjusted hazard ratios (AHR) and 95% CIs were reported. Missing values were estimated from multiple imputations using a chained equation (MICE) algorithm under the assumption of a missing at random (MAR) mechanism [25]. The P-value < 0.05 was considered as significant. The statistical analysis of data was performed using SPSS version 22.

### Ethical approval

The study protocol was reviewed and approved by Ethics Committee of Kerman University of Medical Sciences (reference number: IR.KMU.REC.1398.078). Ten percent of women declined to participate, and other eligible were included to replace them. After explaining the aims of the study, the written informed consent was obtained from the participants and were assured that their information would remain confidential. This study was conducted in compliance with the Helsinki Declaration.

## Results

Table 1 shows the descriptive characteristics of the wives and husbands participated in the study. The majority of wives (67.93%) and husbands (70.37%) had high school education. More than half of the families earned sufficient income (54.3%). The majority of women (60.6%) got married over the age of 20 and most of them did not have any underlying disease (75.48%). Most of the husbands got married between 20 up to 30 years of age (54.96%). The average of FBI was 2.5 ∓ 0.8 years.

**Table 1:** Descriptive characteristic of women (n = 1350) total and based on residence place

Variables				
Women’s predictors	Levels	Total N (%)	Rural 368 (27.26)	Town 982 (72.74)
Women’s education	Illiterate	7 (0.52)	5 (1.35)	2 (0.22)
	High school and Diploma	917 (67.93)	347 (94.29)	570 (61.42)
	University education	426 (31.56)	16 (4.35)	410 (44.18)
Women’s age at marriage (year)	≤ 15	48 (3.6)	12 (3.26)	36 (3.88)
	15–20	483 (35.8)	192 (52.17)	291 (29.63)
	> 20	818 (60.6)	168 (45.65)	655 (66.70)
Women’s drug use	Yes	47 (3.5)	22 (5.97)	25 (2.55)
	No	1303 (96.5)	346 (94.02)	957 (97.45)
Women underlying diseases	Yes	331 (24.52)	98 (26.63)	233 (23.73)
	No	1019 (75.48)	270 (73.37)	749 (76.27)
Night job of women	Yes	231 (17.11)	1 (0.27)	230 (23.42)
	No	1119 (82.89)	367 (99.73)	752 (76.58)
Mean ∓ SD				
Women’s BMI (mean ± SD)		26.53 ± 56.23	25.48 ± 26.21	26.75 ± 35.26
Age at the first menstruation (year)		13.03 ± 1.23	12.98 ± 56.23	13.09 ± 56.23
Husband’s predictors				
Husband’s education	Illiterate	15 (1.12)	12 (3.26)	3 (0.31)
	High school and Diploma	950 (70.37)	220 (59.78)	760 (77.39)
	University education	385 (28.52)	136 (36.95)	219 (22.30)
Husband’s drug use	Yes	208 (15.41)	156 (42.39)	52 (5.29)
	No	1142 (84.59)	212 (57.6)	930 (94.70)
Age at marriage (year)	< 20	41 (3.04)	16 (4.35)	25 (2.54)
	20–30	742 (54.96)	229 (62.23)	513 (52.24)
	> 30	567 (42)	123 (33.42)	444 (45.21)
Night job of husband	Yes	601 (44.52)	115 (31.25)	486 (49.49)
	No	749 (55.48)	253 (68.75)	496 (50.51)
Family predictors				
Income	Sufficient	733 (54.3)	205 (55.71)	528 (53.76)
	Insufficient	617 (45.7)	163 (44.92)	454 (46.23)
Contraception use	Yes	1025 (75.93)	267 (72.55)	758 (77.19)
	No	325 (24.07)	101 (27.44)	224 (22.81)
Having engagement period	Yes	892 (66.07)	114 (30.97)	778 (79.23)
	No	458 (33.92)	254 (69.03)	204 (20.77)
Marriage type	Family	390 (18.52)	214 (58.15)	176 (17.92)
	Not family	1100 (81.48)	154 (41.85)	946 (96.33)

*BMI* Body Mass Index

Table 2 shows the results of univariate (crud) and then the predictors with P-value less than 0.15 were entered into the multivariable model.

**Table 2:** Results of the univariate Cox proportional hazards analysis (crude analysis) to evaluated factors related to FBI

	$\beta$	HR	SE (β)	95% CI for HR	P-value
Women’s predictors					
Women’s age at marriage	− 0.338	1.402	0.421	1.29–2.92	< 0.001
Age at the first menstruation	− 0.461	1.587	0.383	1.14–2.73	0.002
Women’s drug use vs. none	− 0.199	1.221	0.122	0.11–2.04	0.071
Women’s BMI	0.594	1.812	0.484	1.47–2.92	0.001
Women’s underlying disease	− 0.208	1.232	0.118	0.74–1.72	0.111
High school and Diploma vs. illiterate	− 0.167	1.182	0.187	0.45–1.66	0.141
University education vs. illiterate	0.427	1.534	0.323	1.45–2.72	0.005
Contraception use	0.963	2.621	0.254	1.21–2.92	< 0.001
Night job of mother vs. daily	− 0.158	1.172	0.122	0.27–1.91	0.091
Husband’s predictors					
Husband’s age at marriage	− 0.753	2.124	0.014	1.71–2.42	< 0.001
High school and Diploma vs. illiterate	− 0.439	1.552	0.098	0.27–2.14	0.084
University education vs. illiterate	0.423	1.526	0.452	1.41–2.56	0.001
Husband drug use vs. None	0.489	1.632	0.152	0.28–2.21	0.132
Night job of husband vs. daily	− 0.203	1.226	0.221	0.24–1.82	0.142
Family predictors					
Family marriage vs. not family	− 0.196	1.217	0.119	0.32–2.21	0.134
Rural residence vs. town	− 0.942	2.564	0.488	1.37–3.11	0.003
Income sufficiency vs. not sufficient	0.885	2.423	0.158	1.29–3.02	< 0.001
Having engagement period vs. none	− 0.653	1.921	0.045	1.62–3.33	0.002

*HR* hazard ratio; *SE* standard error; *CI* confidence interval

Table 3 shows the results of multiple Cox regression. The sign of beta coefficient shows the direction of predictors effect on FBI. HR shows the rate of each predictor’s effect on FBI. As observed, the women who were one year older at the age of marriage were 1.5 times more likely to have shorter FBI (HR 1.482, 95% CI 1.32, 2.48, P = 0.023). The women who experienced menarche one year older were 1.5 times more likely to have shorter FBI (HR 1.534, 95% CI 1.24, 2.53, P = 0.035). Increasing one unit in women’s BMI increased FBI by 1.73 times (HR 1.729, 95% CI 1.54, 2.77, P = 0.014). University educated women are 1.5 times more likely to have longer FBI (HR 1.473, 95% CI 1.35, 2.55, P = 0.026). Women who used contraception were 2.28 times more likely to have longer FBI (HR 2.337, 95% CI 1.23, 2.76, P < 0.001). Husbands who were one year older at the age of marriage were 1.88 times more likely to have shorter FBI (HR 1.881, 95% CI 1.62, 2.03, P < 0.001). University educated husbands were 1.4 times more likely to have longer FBI (HR 1.392, 95% CI 1.32, 2.54, P = 0.021). Couples who lived in rural areas were 2.04 times more likely to have shorter FBI (HR 2.041, 95% CI 1.26, 2.95, P = 0.031). Family income sufficiency increased FBI by 2.26 times (HR 2.255, 95% CI 1.12, 2.56, P < 0.001). Couples who had engagement period were 1.85 times more likely to have shorter FBI (HR 1.853, 95% CI 1.52, 3.05, P = 0.021).

**Table 3:** Results of the multiple Cox proportional hazards analysis (adjusted analysis) to evaluated factors related to FBI among women

	$\beta$	HR	Se (β)	95% CI for HR	P-value
Women’s predictors					
Women’s age at marriage	− 0.392	1.479	0.357	1.32–2.48	0.023
Age at the first menstruation	− 0.428	1.534	0.329	1.24–2.53	0.035
Women’s drug use vs. none	− 0.013	1.013	0.108	0.16–1.92	0.872
Women’ s BMI	0.548	1.729	0.447	1.54–2.77	0.014
Women’s underlying disease	− 0.012	1.012	0.107	0.87–1.68	0.882
High school and Diploma vs. illiterate	− 0.071	1.074	0.077	0.33–1.59	0.346
University education vs. illiterate	0.387	1.473	0.257	1.35–2.57	0.026
Contraception use	0.822	2.275	0.133	1.12–2.86	< 0.001
Night job of mother vs. daily	− 0.067	1.069	0.097	0.26–1.81	0.368
Husband’s predictors					
Husband’s age at marriage	− 0.632	1.881	0.003	1.62–2.03	< 0.001
High school and Diploma vs. illiterate	− 0.043	1.044	0.071	0.22–1.98	0.624
University education vs. illiterate	0.331	1.392	0.347	1.32–2.51	0.021
Husband drug use vs. None	0.041	1.042	0.055	0.27–1.87	0.488
Night job of husband vs. daily	− 0.013	1.013	0.106	0.23–1.78	0.762
Family predictors					
Family marriage vs. not family	− 0.015	1.015	0.104	0.24–1.91	0.771
Rural residence vs. town	− 0.712	2.041	0.431	1.26–2.95	0.031
Income sufficiency vs. not sufficient	0.813	2.255	0.141	1.12–2.96	< 0.001
Having engagement period vs. none	− 0.617	1.853	0.033	1.52–3.05	0.021

*HR* hazard ratio, *SE* standard error; *CI* confidence interval

## Discussion

Drawing on a large sample, the study indicates that there is a positive association between FBI and age of wives at marriage, first menarche age, wives’ BMI, wives and husbands’ educational level, contraception use, age of husbands at marriage, residence place, income sufficiency, and having engagement period.

Regarding the social-demographic variables, the women and men who got married at older ages were more likely to report short FBI. This finding is in accordance with the result of several studies such as Nigeria, Iran, and south of Iran respectively [19, 26, 27]. Moreover, a similar study conducted in India demonstrated positive association between increase in couple’s age at marriage and short FBI [7]. This association could be explained by reproductive age limit, especially for women. The women who get married at a later age have few reproductive years, so they want to have the first child sooner [8, 28].

Couples' university education was found to be a significant predictor of FBI. It was indicated that educated women and men are more likely to have longer FBI. This finding is consistent with other studies like a cross-sectional study conducted in Indonesia (2018), a study conducted in Iran (2019), and a cross-sectional study conducted in China (1996) [4, 8, 29]. Similarly, a study in Nigeria indicates that education delays marriage, and the maturity that comes with age may result in more effective contraception, hence influences the birth interval. Education increases the employment opportunity in the modern sector, and this competes with the demand for childbearing. Through increasing her odds of being employed outside home and thus becoming an income-producing member of the family, a woman is more likely to acquire a role in decision makings concerning all aspects of family life, including the number and spacing of her children [7, 26].

This study also demonstrated a significant association between FBI and the first menstrual age. It was indicated that women who experience menstruation at a younger age are more likely to report longer FBI. These findings are consistent with those of the previous study [30]. Maybe it's because girls who had menstruation at a younger age got married earlier, but because they were young at the time of the marriage, they avoided getting pregnant, so they got pregnant later. Or maybe it was because of hormonal disorders that she had an earlier menstruation and these hormonal disorders caused her to have children later. The rural residents are more likely to have shorter FBI. This finding was consistent with that of the previous study indicating that rural couples desire more children to assist with agricultural and other work. People living in the rural areas desired to have more children with short birth spacing in order to have more assistants [4, 19, 31]. The authors of the present study also documented the role of engagement duration in decreasing FBI. This may be caused due to couples familiarity increase during engagement. These couples decide to have a child sooner.

In-line with previous research [32] the present researchers' findings also support the positive role of high BMI of women on increasing FBI. This positive association could be due to the role of obesity on decreasing fertility. Research has shown that obesity in women increases FBI. Obesity is associated with polycystic ovary syndrome, which is characterized by oligo or lack of ovulation, hyperandrogenism, menstrual irregularities, and infertility [33, 34]. Obese women may desire to have their first child sooner, but high weight has a deterrent role.

The present researchers observed that the families with income sufficiency tend to have their first child later similar to another study conducted in rural areas of Shiraz [15]. Another study conducted in Bangladesh had consistent findings with those of the present study and revealed that high family economic status leads to long birth spacing [31].

In-line with the study in Nigeria, contraception use was associated with increased FBI [35]. Previous studies have also showed a strong link between contraception use and birth spacing [36, 37].

### Limitations

The present study has several limitations: First, a cross-sectional type of study cannot prove causality. The effect of factors could be explored across a longitudinal study in order to show the significance of predictors with more precision. Second, many factors such as biological, economic, social, and cultural factors influence the time of the first birth, and the present researchers had budget and time limitation to involve all of them. The role of more biological predictors could be explored in future studies.

## Conclusion

Being a rural resident is the most influential predictor of short first-birth interval, and income sufficiency is the most influential predictor of long first-birth interval. The knowledge about importance of FBI should be more announced for couples by health services in order to help them manage their child-spacing and fertility.

## Data Availability

The datasets generated during and/or analyzed during the current study are available from the corresponding author on reasonable request.

## Abbreviations

FBI: First-birth interval
95% CI: 95% Confidence interval
BP: Blood pressure
BMI: Body mass index
SD: Standard deviation
AHR: Adjusted hazard ratios
HR: Hazard ratios
MICE: Multiple imputations using a chained equation
MAR: Missing at random
